# Effects of intradialytic nutrition on dialysis adequacy and fatigue in hemodialysis patients: a randomized crossover study

**DOI:** 10.1590/1980-220X-REEUSP-2026-0010en

**Published:** 2026-09-11

**Authors:** Esra Köse, Meral Gün

**Affiliations:** 1 Mersin University Hospital, Faculty of Medicine, Hemodialysis Unit, Mersin, Türkiye.; 2 Mersin University, Faculty of Nursing, Department of Internal Medicine Nursing, Mersin, Türkiye.

**Keywords:** Renal Dialysis, Eating, Fatigue, Kinetics., Diálise Renal, Ingestão de Alimentos, Fadiga, Cinética.

## Abstract

**Objective:**

To assess intradialytic nutrition’s effects on dialysis adequacy and fatigue in maintenance hemodialysis patients.

**Methods:**

A randomized, two-period, two-sequence, self-controlled crossover trial was conducted in two outpatient hemodialysis centers in Türkiye. Thirty-six patients were randomized; 32 completed both periods. The participants received standardized intradialytic nutrition in one period and no food intake during the control period. Dialysis adequacy was evaluated using the urea reduction ratio and single-pool Kt/V. Intradialytic blood pressure was recorded during dialysis sessions, and fatigue severity was measured using the Piper Fatigue Scale at the end of each period.

**Results:**

No significant differences were observed in dialysis adequacy or fatigue scores between the two periods. Intradialytic nutrition was associated with greater reductions in systolic and diastolic blood pressure, particularly during the second hour of dialysis. No clinically relevant adverse events occurred.

**Conclusion:**

Intradialytic nutrition did not compromise dialysis adequacy or worsen fatigue severity but was associated with increased intradialytic blood pressure reductions. Individualized clinical decisions and careful hemodynamic monitoring are warranted when implementing intradialytic nutritional interventions.

**Trial Registration:**

ClinicalTrials.gov Identifier: NCT07687498.

## RESUMO

**Objetivo:**

Avaliar os efeitos da alimentação intradialítica sobre a adequação da diálise e a fadiga em pacientes em hemodiálise de manutenção.

**Métodos:**

Ensaio clínico randomizado, cruzado, com dois períodos, duas sequências e autocontrole, realizado em dois centros ambulatoriais de hemodiálise na Turquia. Trinta e seis pacientes foram randomizados e 32 completaram ambos os períodos do estudo. Os participantes receberam alimentação intradialítica padronizada em um período e permaneceram em jejum durante a diálise no período controle. A adequação da diálise foi avaliada pela taxa de redução da ureia (URR) e pelo Kt/V de compartimento único (spKt/V). A pressão arterial intradialítica foi registrada durante as sessões de diálise e a intensidade da fadiga foi avaliada pela Escala de Fadiga de Piper ao final de cada período.

**Resultados:**

Não foram observadas diferenças significativas entre os períodos de intervenção e controle quanto à adequação da diálise ou aos escores de fadiga. A alimentação intradialítica esteve associada a maiores reduções da pressão arterial sistólica e diastólica, especialmente durante a segunda hora da diálise. Não ocorreram eventos adversos clinicamente relevantes.

**Conclusão:**

A alimentação intradialítica não comprometeu a adequação da diálise nem a intensidade da fadiga, porém esteve associada a maiores reduções da pressão arterial durante a diálise. Recomenda-se tomada de decisão clínica individualizada e monitorização hemodinâmica cuidadosa ao implementar intervenções nutricionais intradialíticas.

## INTRODUCTION

Chronic kidney disease (CKD) is becoming a growing burden on global health, and hemodialysis (HD) remains the most commonly used renal replacement therapy for patients with end-stage renal disease^(1,2,3)^. Despite technological advances in dialysis treatments, HD patients continue to experience a high burden of symptoms during dialysis, such as hypotension, fatigue, and nutritional deficiencies, all of which negatively impact their quality of life and clinical outcomes^(4,5,6)^.

Dialysis adequacy, commonly assessed using the urea reduction ratio (URR) and single-pool Kt/V (spKt/V), remains a cornerstone of the quality evaluation of HD^(7,8,9)^. However, the potential impact of food intake during dialysis sessions on solute clearance and treatment effectiveness continues to be debated. Recent studies have suggested that intradialytic nutrition may improve nutritional status and patient-centered outcomes, particularly in individuals at risk of protein-energy loss^(6,10,11)^. Conversely, concerns persist regarding its potential to impair dialysis adequacy and increase the risk of intradialytic hypotension due to postprandial hemodynamic changes^(8,9,12,13,14)^.

Existing evidence emphasizes that the effects of food intake during hemodialysis are highly variable and may depend on factors such as meal composition, timing, patient characteristics, and dialysis prescription. Some studies have reported no clinically significant impact on URR or Kt/V when meals were standardized^(6,7,13,14)^, whereas others have suggested that the redistribution of blood flow following food intake may affect solute clearance^(8,15,16)^.

Furthermore, international guidelines and expert consensus statements offer conflicting recommendations. According to the International Society of Renal Nutrition and Metabolism^(17)^, as well as recent narrative and systematic reviews, nutrition during dialysis cannot be universally recommended and must instead be tailored to a patient’s specific hemodynamic tolerance and metabolic status^(6,18,19)^. Evidence regarding the effect of nutrient intake during dialysis on dialysis adequacy is inconsistent. Although several studies have reported a decrease in URR and spKt/V associated with food consumption during HD sessions^(8,9)^, others have found no clinically meaningful effect, particularly when meals were standardized and of limited volume^(7,13,20)^. These inconsistencies may result from heterogeneity in study designs, meal composition, dialysis prescriptions, and patient characteristics.

In addition to dialysis adequacy, hemodynamic stability during dialysis is a critical clinical concern. Postprandial hypotension during HD is attributed to a combination of increased splanchnic blood flow, reduced systemic vascular resistance, and impaired compensatory cardiovascular responses^(8,12,18,20)^. Intradialytic hypotension is associated with increased morbidity, prolonged recovery time, and reduced patient well-being. Therefore, understanding the hemodynamic consequences of intradialytic eating is essential.

Fatigue is one of the most common symptoms reported by HD patients. Post-HD fatigue, which can last for hours and does not fully resolve with rest, significantly affects the daily activities, treatment participation, and quality of life of patients. Studies have shown that 60%–97% of HD patients experience fatigue, which significantly impacts their social life and quality of life^(4,5,6,21)^. Recent research has highlighted that fatigue is a multifaceted symptom affected by several factors, such as inflammation, comorbidities, sleep disturbances, nutritional status, and the HD procedure itself. Contributing factors include the accumulation of metabolic waste (urea, uric acid, and creatinine), inadequate dialysis, fluid restrictions, blood pressure fluctuations, excessive ultrafiltration, electrolyte imbalance, anemia, and psychological factors. Inactivity during treatment may also lead to muscle weakness, further increasing fatigue. In addition, uremic symptoms such as nausea, vomiting, and loss of appetite can cause malnutrition, which exacerbates fatigue^(5,6,22)^. Although patient-reported outcome measures are increasingly emphasized in nephrology research, no study has directly examined the relationship between intradialytic food intake and fatigue severity; this represents a significant gap in the literature. Therefore, the present randomized, self-controlled crossover study aimed to evaluate the effects of intradialytic nutrition on dialysis adequacy, intradialytic hemodynamic parameters, and fatigue in maintenance HD patients. The research objective is to provide clinical evidence regarding individualized intradialytic nutrition practices by integrating objective dialysis measurements with patient-reported outcomes. Accordingly, the hypothesis that intradialytic food intake has no effect on dialysis adequacy (URR and spKt/V) and fatigue levels was tested.

## METHOD

### Design of Study

This study was designed as a randomized, two-period, two- sequence crossover trial in which each participant served as their own control. The crossover methodology was implemented in accordance with considerations regarding crossover design in clinical trials^(23)^. Furthermore, this manuscript was prepared according to the Consolidated Standards of Reporting Trials (CONSORT) 2010 extension for randomized crossover trials^(24)^.

### Population and Selection Criteria

Adult patients undergoing HD at a university hospital and a private dialysis center in Türkiye were screened for eligibility. The inclusion criteria were as follows: age ≥18 years, HD received at least three times per week for a minimum of three months, stable clinical condition, and ability to provide informed consent. The exclusion criteria were as follows: active infection, hospitalization within the previous month, gastrointestinal disorders affecting oral intake, severe intradialytic hypotension requiring frequent intervention (MAP <65 mmHg), cognitive impairment, pregnancy, and upcoming scheduled kidney transplantation due to the availability of a suitable donor during the study period.

### Sample Size

The study population comprised 127 patients, 25 of whom were treated at the university hospital and 102 at the dialysis center. The sample size was calculated based on the study by Kara and Açıkel^(25)^. Accordingly, with an effect size (d) of 0.71, a type I error of 0.05, a power of 0.80, and a correlation value between groups of 0.50, it was determined that at least 18 patients should be included^(26)^. However, considering the possibility of patients dropping out for any reason, a dropout rate of 50 was added, and it was determined that at least 36 patients were needed as the initial sample. Ultimately, a total of 36 patients were randomized; of these, 32 completed both study periods and were included in the final analysis.

### Randomization and Blinding

The participants were randomly assigned to one of two intervention sequences in a 1:1 ratio using a computer-generated random assignment list. Sequence A entailed dialysis-based nutrition in the first period, followed by no nutrition in the second period; Sequence B involved no nutrition in the first period, followed by in-dialysis nutrition in the second period. The random assignment was performed by an independent researcher not involved in the data collection process. Due to the nature of this research, the participants and the researchers could not be blinded.

### Intervention

During the nutrition period in dialysis, the participants consumed a standardized meal one and a half hours after the start of dialysis. The meal provided approximately 450 kcal and 20 g of protein, with low potassium and phosphorus content, in accordance with renal dietary recommendations^(14,17)^. Sample menu contents included one boiled egg, one slice of white cheese, six unsalted black olives or five green olives, one slice of unsalted bread, one 20-g portion of strawberry or apricot jam, and 200 mL of black tea. The participants were instructed to consume the entire meal and not to consume any additional food during dialysis. During the control period, the participants did not consume any food during dialysis sessions but continued their usual diet outside of dialysis. Each study period lasted four weeks, corresponding to approximately 12 dialysis sessions per period.

Regarding the dialysis procedure, the HD parameters—namely, four hours three times a week, Fresenius 4008s machine, Renal-brand glucose-containing dialysate solution, Fresenius-brand synthetic high-flux dialyzer (1.7 m^2^), dialysate sodium concentration of 140 mEq/L, blood flow rate (300–350 ml/min), dialysate flow rate (500 ml/min), and dialysate temperature (36–36.5°C)—were kept constant.

### Outcome Measures

**Figure 1:**
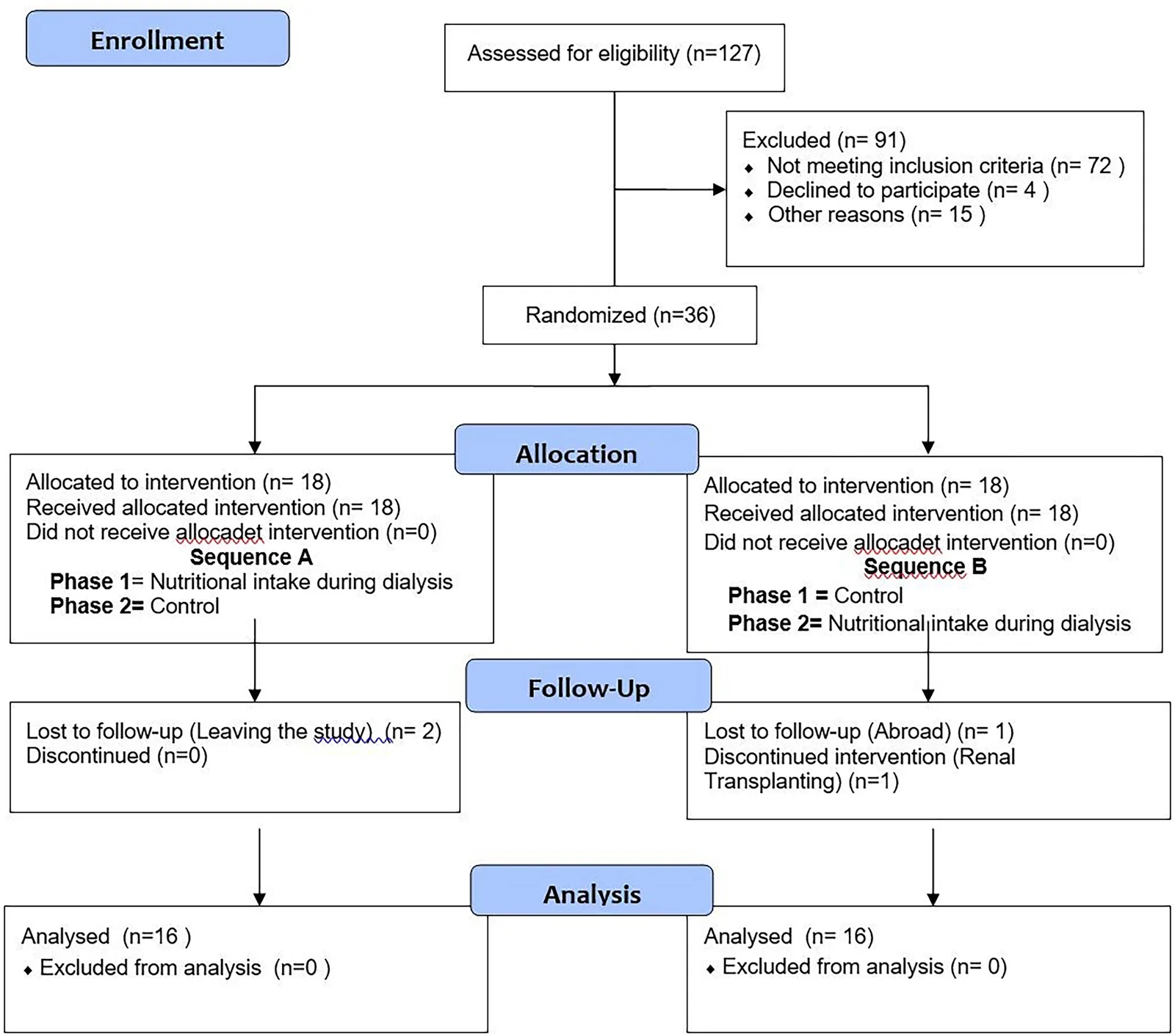
Flowchart of the participants included and analyzed in the study, in compliance with the Consolidated Standards of Reporting Trials (CONSORT) – Mersin, Türkiye.

The primary outcomes were dialysis adequacy, assessed using the URR and spKt/V calculated from routine pre- and post-dialysis blood samples, and fatigue severity, which was measured at the end of each study period. The secondary outcomes comprised intradialytic systolic blood pressure (SBP) and diastolic blood pressure (DBP), both measured at baseline and hourly during dialysis; the incidence of intradialytic hypotension; ultrafiltration volume; and laboratory parameters. This study design is summarized in Figure 1.

### Data Collection

Data were collected between April 1, 2022, and June 30, 2022, by one of the researchers (EK), who has 25 years of experience as a specialist nurse in a university hospital HD unit and has actively participated in various courses, trainings, and scientific congresses related to HD. Data collection was carried out using three instruments: the Personal Information Form, the Hemodialysis Monitoring and Laboratory Findings Form, and the Piper Fatigue Scale (PFS).

The Personal Information Form, developed based on the literature, comprised 15 items assessing sociodemographic characteristics (e.g., age, sex, marital status, and educational status) and disease-related variables (e.g., etiology of renal failure, comorbidities, antihypertensive use, medications, and symptoms experienced)^(7,14,17)^.

The Hemodialysis Monitoring and Laboratory Findings Form was also developed based on existing research^(7,8)^. It was used to gather data on intradialytic blood pressure measurements (pre-dialysis, at the second hour, and post-dialysis), ultrafiltration volume, dry weight, and pre- and post-dialysis body weight. Routine monthly laboratory parameters (urea, creatinine, potassium, phosphorus, albumin, and hemoglobin), as well as dialysis adequacy indices (spKt/V and URR), were also recorded^(10,11,25)^.

Fatigue was assessed using the PFS, a 22-item instrument developed by Piper et al.^(27)^. Total fatigue scores were calculated as the mean of item scores, with higher scores indicating greater perceived fatigue. The Turkish validity and reliability study for this scale was conducted by Can et al.^(28)^, who reported a Cronbach’s alpha of 0.94^(28)^. In the present study, the Cronbach’s alpha coefficient was 0.92.

### Data Analysis and Treatment

The collected data were analyzed using statistical software (IBM SPSS version 25). Continuous variables were tested for normality using the Shapiro–Wilk test. Paired t tests and repeated-measures analysis of variance (ANOVA) were used to compare two and three related measurements, respectively, with Bonferroni adjustment applied for post hoc comparisons. Continuous data are presented as means and standard deviations, and categorical data are presented as frequencies and percentages. Pearson’s correlation coefficient was used to examine associations between continuous variables. Statistical significance was set at p < 0.05.

### Ethical Aspect

Ethical approval was obtained from the Clinical Research Ethics Committee of the relevant institution (approval no. 199, dated March 23, 2022), as well as from the hospital and the dialysis center involved in this study. Written and verbal informed consent was obtained from all participants after they were fully informed about the study purpose and procedures. Furthermore, this study was conducted in accordance with the principles of the Declaration of Helsinki. This study’s ClinicalTrials.gov identifier is NCT07687498.

## RESULTS

The average age of the participants was 53.7 ± 16.8 years, and the majority were 60 years or older. Hypertension was the most common cause of CKD, and most participants had at least one additional chronic condition. Fatigue was the most frequently reported symptom (84.4%), followed by sleeplessness (62.5%) and weakness (56.3%).

### Laboratory, Dialysis Adequacy and Ultrafiltration Volume

The analysis of laboratory parameters indicated no statistically significant differences between the intradialytic eating and noneating periods, with the exception of serum phosphorus levels (p > 0.05). Specifically, serum phosphorus concentrations were significantly higher during the intradialytic eating period (p = 0.02). Similarly, no statistically significant differences were observed in dialysis adequacy parameters, including URR, spKt/V, and ultrafiltration volume, between the two periods (p > 0.05) (Table 1).

**Table 1:** Comparison of laboratory findings and URR–spKt/V values between patients with and without food intake during dialysis – Mersin, Türkiye, 2022 (n = 32).

Variables	With food intake Mean ± SD	Without food intake Mean ± SD	Test t	Significance p
Potassium (mEq/L)	5.54 ± 0.68	5.36 ± 0.72	1.278	0.21
Albumin (gr/dl)	3.76 ± 0.42	3.74 ± 0.42	0.579	0.56
Phosphorus (mgr/dl)	5.92 ± 1.67	5.28 ± 1.86	2.323	0.02
Hemoglobin (gr/dl)	10.44 ± 2.83	10.19 ± 2.89	0.766	0.44
URR	73.21 ± 6.37	73.43 ± 5.88	-0.217	0.83
spKt/V	1.60 ± 0.3	1.61 ± 0.25	-0.327	0.75
**Ultrafiltration quantity (UF)**				
Entry-exit weight difference	2584.38 ± 683.96	2712.5 ± 665.15	-1.339	0.19

Paired t-test, p < 0.05 significance.

### Blood Pressure

The overall analysis of intradialytic blood pressure values showed a significant progressive decrease in SBP during sessions with food intake compared with those without food intake (p = 0,001). Mean SBP at the second hour of dialysis was significantly lower during the eating period (118.72 ± 15.68 vs. 122.59 ± 16.22; p = 0.04). Similarly, DBP values at the second and third measurements declined significantly during the eating period (p = 0,001), whereas the decreases observed during the noneating period were not statistically significant (p > 0.05) (Table 2).

**Table 2:** Comparison of blood pressure values between patients with and without food intake during dialysis – Mersin, Türkiye, 2022 (n = 32).

Variables	With food intake Mean ± SD	Without food intake Mean ± SD	Test t	Significance P*
**Systolic blood pressure**				
Dialysis Input SBP	127.63 ± 13.87	127.69 ± 13.89	-0.056	0.96
2nd-hour SBP	118.72 ± 15.68	122.59 ± 16.22	-2.089	0.04
Dialysis Outflow SBP	116.94 ± 17.56	116.94 ± 17.56	-1.081	0.29
F	23.610	20.983		
P**	<0.001	<0.001		
***1 vs 2, 1 vs 3, 2 vs 3	<0.001; < 0.001; 0.41	0.002; <0.001; 0.02		
**Diastolic blood pressure**				
Dialysis Input DBP	80.41 ± 10.48	80.63 ± 13.43	-0.136	0.89
2nd hour DBP	75.31 ± 10.52	76.97 ± 10.88	-1.311	0.2
Dialysis Outflow DBP	73.41 ± 12.65	76.41 ± 15.08	-1.212	0.24
F	23.916	2.446		
P**	<0.001	0.1		
***1 vs 2, 1 vs 3, 2 vs 3	<0.001; <0.001; 0.04	–		

*Paired t test,

**Repeated ANOVA,

***Post Hoc Test Bonferroni,

p < 0.05 significance.

### Fatigue

When the periods with and without food intake were compared, no statistically significant differences were found between the groups in terms of the definition of fatigue (tiredness, weakness, and mental fatigue), its causes, or coping strategies (p > 0.05). HD sessions were reported as the most common cause of fatigue in both periods and were more frequently reported during the noneating period; however, the difference was not statistically significant (p > 0.05). With regard to managing fatigue, most participants reported engaging in passive or low-intensity activities after dialysis, such as resting or sleeping, watching television, and walking (Table 3). The mean PFS scores were 106.88 ± 27.51 during the intradialytic eating period and 108.63 ± 29.36 during the noneating period, with no statistically significant differences observed between the two conditions (p > 0.05) (Table 4).

**Table 3:** Symptoms experienced by patients, coping approaches, and intergroup comparisons – Mersin, Türkiye, 2022 (n = 32).

Variables	With food intaken (%)	Without food intaken (%)	Statisticsx2 test/p-value
Fatigue definition*			
Tiredness	12 (57%)	9 (43%)	0.384/0.54
Weakness	12 (50%)	12 (50%)	<0.001/0.999
Mental fatigue	9 (41%)	13 (59%)	0.659/0.42
Causes of fatigue*			
Hypotension	9 (60%)	6 (40%)	0.538/0.46
Hemodialysis session	10 (37.01%)	17 (62.9%)	1.633/0.20
Conditions related to anemia	3 (37.5%)	5 (62.5%)	0.412/0.52
Hemodialysis transportation vehicle	11 (61.1%)	7 (38.9%)	0.789/0.37
Chronic pain	2 (33.3%)	4 (66.6%)	0.499/0.48
How to cope with the symptom of fatigue*			
Sleeping/resting	13 (65%)	7 (35%)	1.569/0.21
Taking a walk	10 (55.5%)	8 (44.5%)	0.203/0.65
Watching TV/Listening to Music	13 (61.9%)	8 (38.1%)	1.072/0.30
Working with plants	4 (33.3%)	8 (66.6%)	1.098/0.29
Traveling with friends or family	5 (55.5%)	4 (44.4%)	0.097/0.76
Caring for Animals	3 (33.33%)	6 (66.6%)	0.798/0.37
Reading	8 (57.14%)	6 (42.85%)	0.260/0.61

*More than one option ticked.

**Table 4:** Comparison of Piper Fatigue Scale (PFS) mean scores between patients with and without food intake during dialysis – Mersin, Türkiye, 2022 (n = 32).

Variables	With food intake Mean ± SD	Without food intake Mean ± SD	Test t	Significance P
PFS	106.88 ± 27.51	108.63 ± 29.36	-0.409	0.69

Paired t-test, p < 0.05 significance.

## DISCUSSION

In this randomized crossover study, intradialytic nutritional intake did not significantly affect dialysis adequacy parameters, including URR and spKt/V, nor did it alter fatigue severity. However, intradialytic nutrition was associated with a more pronounced decrease in SBP and DBP during dialysis sessions, particularly during the middle phase of treatment. These findings provide clinically relevant information regarding both the potential benefits and risks of food intake during HD and support a more individualized approach to intradialytic nutritional practices.

In this study, serum phosphorus levels were found to be significantly higher when food was consumed during HD. Previous studies have reported that intradialytic nutrition may contribute to hyperphosphatemia due to the phosphorus content of meals^(11,20)^. However, in the present study, phosphorus levels remained within acceptable clinical limits, likely due to the standardized meal composition and concurrent phosphate-binding therapy. This finding suggests that carefully planned meals with appropriate phosphorus restriction may reduce potential metabolic risks when intradialytic nutrition is implemented.

The results of this study indicate that intradialytic nutrient intake does not compromise dialysis adequacy. This finding is consistent with previous studies reporting minimal or clinically nonsignificant changes in URR or spKt/V when meals were standardized and dialysis prescriptions remained unchanged^(6,7,10,11,13)^. Rao et al. demonstrated that intradialytic nutrition did not significantly reduce urea removal compared with predialytic eating, particularly when meal composition and timing were controlled^(7)^. Setiovati et al. stated that the most appropriate time for intradialytic eating is the late phase of the dialysis session. Specifically, they reported that eating in the first and second hours led to more significant hemodynamic changes in SBP and MAP, whereas eating toward the end of the session was associated with a more stable hemodynamic response and better preservation of dialysis adequacy. However, some studies have reported that food consumption during dialysis may reduce dialysis adequacy; therefore, they do not recommend intradialytic eating^(8,18,20)^. Kara and Açıkel^(25)^ found a significant decrease in dialysis adequacy parameters among patients consuming food during dialysis, suggesting that splanchnic vasodilation and altered blood flow distribution may impair solute clearance. These inconsistencies in existing research may be explained by differences in meal size, macronutrient composition, and dialysis technology. Importantly, the present study utilized a self-controlled crossover design, which minimizes inter-individual variability and enhances internal validity. Furthermore, the participants had a relatively high baseline dialysis competency, which might have limited the potential to detect small decreases in URR or spKt/V. These findings support the increasingly prevalent view that dialysis adequacy measures alone may not fully reflect the clinical impact of intradialytic interventions on splanchnic vasodilation^(10,11,12,13)^. As patients spend a long time in HD sessions, allowing them to choose their favorite foods can make these sessions more bearable for them and improve their treatment adherence^(13,14,29)^. The most appropriate approach would be to address patients’ nutritional status with individualized strategies based on their risk factors, especially for patients prone to autonomic neuropathy and symptomatic hypotension, in consultation with a dietitian.

The most notable clinical finding in this study was the greater reduction in blood pressure observed during the eating period. The literature contains conflicting results regarding the effect of intradialytic nutrition on intradialytic hypotension. Some studies have reported that intradialytic nutrition may increase hemodynamic instability^(8,12,15,16,18,19)^. However, in other studies, there was no significant increase in the risk of hypotension when appropriate patient selection and controlled nutrition practices were present^(9,10,11,12,13,14,15,22)^. To illustrate, Hou et al.^(30)^ stated that intradialytic hypotension is associated with a low level of physical function and that physical performance is more affected in patients experiencing frequent hypotension; in contrast, Usakli et al.^(31)^ reported that intradialytic nutrition increased hand grip strength and serum albumin levels, while Choi et al. showed that high-protein meals did not increase the frequency of symptomatic intradialytic hypotension and that patients had a positive attitude toward nutrition during dialysis^(13)^. These findings suggest that, despite the potential hemodynamic risks of intradialytic nutrition, it can provide nutritional and functional benefits as long as appropriate patient selection and close monitoring are ensured. From a nursing standpoint, these findings underscore the importance of vigilant intradialytic blood pressure monitoring, individualized risk assessment, and careful patient selection when implementing intradialytic nutritional interventions.

Fatigue remained highly prevalent (84%) in this study, and most of the participants identified the HD procedure itself as a major contributor. Many participants reported coping with fatigue after dialysis by resting, sleeping, or engaging in low-intensity activities. These findings are consistent with those of previous studies^(4,5,6,21,22,29)^, showing that fatigue is one of the most burdensome symptoms among patients receiving maintenance HD. You et al.^(32)^ found that 71.3% of the patients in their study experienced fatigue. In Bossola et al.’s study^(5)^, emotional symptoms (feeling sad, feeling anxious, and struggling to concentrate) were significantly higher in fatigued patients. Casaux-Huertas et al.^(22)^ reported that poor nutritional status in HD patients is associated with fatigue and sleep disturbances and emphasized that adequate nutritional support, especially intradialytic nutrition applications, may provide potential benefits in terms of reducing symptom burden and improving quality of life. However, intradialytic nutrition did not significantly change fatigue scores. Fatigue in HD patients is a complex and multifactorial symptom influenced by anemia, inflammation, sleep disturbances, comorbid conditions, depression, dialysis-related stress, and psychosocial factors^(4,5,6,22,30,32)^. Therefore, a short-term nutritional intervention alone may be insufficient to produce measurable improvements in fatigue severity. Nevertheless, the absence of worsening fatigue suggests that intradialytic nutrition may be tolerated from a symptom perspective in appropriately selected patients.

Overall, the current findings support a personalized approach to intradialytic nutrition rather than universal restriction or routine implementation. For clinically stable patients with poor nutritional intake, carefully planned meals during dialysis may be beneficial. In contrast, patients with recurrent hypotension, autonomic dysfunction, or significant cardiovascular instability may require more cautious management. Collaboration among nurses, nephrologists, and renal dietitians is essential for optimizing patient safety and nutritional benefit^(6,7,10,14,15,16,17)^. Moreover, these results are consistent with recent expert recommendations advocating individualized decision-making regarding food intake during HD sessions rather than universal policies.

Several limitations of this study should also be acknowledged. First, the relatively small sample size might have limited the statistical power to detect subtle differences in dialysis adequacy and fatigue outcomes. Second, although the crossover design reduced inter-individual variability, the absence of a washout period might have introduced residual carryover effects; however, the acute physiological effects of intradialytic food intake are generally short-lived. Third, as fatigue was assessed using a self-reported instrument, subjective perception and response bias might have influenced the data gathered. In addition, this study was conducted in two centers using a standardized meal protocol, which may limit the generalizability of the findings to other settings with different patient characteristics or nutritional practices. Finally, the relatively short follow-up period restricts conclusions regarding the long-term effects of intradialytic nutrition. Therefore, the findings should be interpreted with these considerations in mind.

## CONCLUSION

This randomized crossover study demonstrated that intradialytic nutrition did not significantly compromise dialysis adequacy or worsen fatigue severity in patients undergoing maintenance HD. However, greater reductions in SBP and DBP were observed following food intake during dialysis, emphasizing the importance of close hemodynamic monitoring during nutritional administration. These findings contribute to the literature by suggesting that intradialytic nutrition may be a clinically feasible approach when tailored to individual patient tolerance and risk profiles, as opposed to being universally restricted or routinely recommended. Importantly, this study also provides novel insights into the relationship between intradialytic nutrition and fatigue—an area that remains insufficiently investigated. From a clinical perspective, the findings highlight the importance of multidisciplinary collaboration among nurses, nephrologists, and renal dietitians when developing nutritional strategies for dialysis sessions. Nevertheless, larger multicenter studies evaluating different nutrient composition types and long-term outcomes are needed to further clarify the safety and clinical impact of intradialytic nutrition.

## Data Availability

The entire dataset supporting the results of this study was published in the article itself.
