# Supplementary material for: Effects of intradialytic nutrition on dialysis adequacy and fatigue in hemodialysis patients: a randomized crossover study
Source: Rev Esc Enferm USP. 2026 Sep 11;60:e20260010. doi: 10.1590/1980-220X-REEUSP-2026-0010en (PMC13577152; doi:10.1590/1980-220X-REEUSP-2026-0010en)
Supplement: Translation

# Translation: Efectos de la nutrición intradiálisis sobre la adecuación de la diálisis y la fatiga en pacientes en hemodiálisis: un estudio cruzado aleatorizado

**DOI:** 10.1590/1980-220X-REEUSP-2026-0010es

**Authors:** Esra Köse, Meral Gün

**Affiliations:** 1 Mersin University Hospital, Faculty of Medicine, Hemodialysis Unit, Mersin, Türkiye.; 2 Mersin University, Faculty of Nursing, Department of Internal Medicine Nursing,, Mersin, Türkiye.

**Keywords:** Diálisis Renal, Ingestión de Alimentos, Fatiga, Cinética.

## Abstract

**Objetivo:**

Evaluar los efectos de la nutrición intradiálisis sobre la adecuación de la diálisis y la fatiga en pacientes en hemodiálisis de mantenimiento.

**Métodos:**

Se realizó un ensayo clínico aleatorizado, cruzado, de dos períodos, dos secuencias y autocontrolado en dos centros ambulatorios de hemodiálisis de Turquía. Se aleatorizaron 36 pacientes y 32 completaron ambos períodos del estudio. Los participantes recibieron nutrición intradiálisis estandarizada durante un período y no consumieron alimentos durante el período control. La adecuación de la diálisis se evaluó mediante la tasa de reducción de urea (URR) y el Kt/V de compartimento único (spKt/V). La presión arterial intradiálisis se registró durante las sesiones de diálisis y la gravedad de la fatiga se evaluó mediante la Escala de Fatiga de Piper al final de cada período.

**Resultados:**

No se observaron diferencias significativas entre los períodos de intervención y control en cuanto a la adecuación de la diálisis ni a las puntuaciones de fatiga. La nutrición intradiálisis se asoció con mayores disminuciones de la presión arterial sistólica y diastólica, especialmente durante la segunda hora de la diálisis. No se produjeron eventos adversos clínicamente relevantes.

**Conclusión:**

La nutrición intradiálisis no comprometió la adecuación de la diálisis ni la gravedad de la fatiga, pero se asoció con mayores descensos de la presión arterial durante la diálisis. Se recomienda la toma de decisiones clínicas individualizadas y una monitorización hemodinámica cuidadosa al implementar intervenciones de nutrición intradiálisis.

## INTRODUCCIÓN

La enfermedad renal crónica (ERC) se está convirtiendo en una carga cada vez mayor para la salud mundial, y la hemodiálisis (HD) sigue siendo la terapia de reemplazo renal más utilizada para los pacientes con enfermedad renal en etapa terminal^(1,2,3)^. A pesar de los avances tecnológicos en los tratamientos de diálisis, los pacientes en HD siguen experimentando una elevada carga de síntomas durante la diálisis, tales como hipotensión, fatiga y deficiencias nutricionales, los cuales afectan negativamente su calidad de vida y sus resultados clínicos^(4,5,6)^.

La adecuación de la diálisis, que se evalúa comúnmente mediante el índice de reducción de urea (IRU) y el Kt/V de un solo compartimento (spKt/V), sigue siendo un pilar fundamental de la evaluación de la calidad de la HD^(7,8,9)^. Sin embargo, el impacto potencial de la ingesta de alimentos durante las sesiones de diálisis sobre el aclaramiento de solutos y la eficacia del tratamiento sigue siendo objeto de debate. Estudios recientes han sugerido que la nutrición intradialítica podría mejorar el estado nutricional y los resultados centrados en el paciente, particularmente en personas con riesgo de pérdida proteico-energética^(6,10,11)^. Por el contrario, persisten las preocupaciones respecto a su potencial para afectar negativamente la adecuación de la diálisis y aumentar el riesgo de hipotensión intradialítica debido a cambios hemodinámicos posprandiales^(8,9,12,13,14)^.

La evidencia existente destaca que los efectos de la alimentación intradialítica son muy variables y pueden depender de factores como la composición de la comida, el momento en que se ingiere, las características del paciente y la prescripción de diálisis. Algunos estudios no han reportado un impacto clínicamente significativo en la URR o el Kt/V cuando las comidas se estandarizaron^(6,7,13,14)^, mientras que otros han sugerido que la redistribución del flujo sanguíneo tras la ingesta de alimentos puede afectar la eliminación de solutos^(8,15,16)^.

Además, las guías internacionales y las declaraciones de consenso de expertos ofrecen recomendaciones contradictorias. Según la Sociedad Internacional de Nutrición y Metabolismo Renal^(17)^, así como revisiones narrativas y sistemáticas recientes, la nutrición durante la diálisis no puede recomendarse de manera universal y, en cambio, debe adaptarse a la tolerancia hemodinámica y al estado metabólico específicos de cada paciente^(6,18,19)^. La evidencia sobre el efecto de la ingesta de nutrientes durante la diálisis en la adecuación de la diálisis es inconsistente. Aunque varios estudios han reportado una disminución de la URR y del spKt/V asociada con el consumo de alimentos durante las sesiones de HD^(8,9)^, otros no han encontrado ningún efecto clínicamente significativo, particularmente cuando las comidas eran estandarizadas y de volumen limitado^(7,13,20)^. Estas inconsistencias pueden deberse a la heterogeneidad en los diseños de los estudios, la composición de las comidas, las prescripciones de diálisis y las características de los pacientes.

Además de la adecuación de la diálisis, la estabilidad hemodinámica durante la diálisis es una preocupación clínica fundamental. La hipotensión posprandial durante la HD se atribuye a una combinación de aumento del flujo sanguíneo esplácnico, reducción de la resistencia vascular sistémica y respuestas cardiovasculares compensatorias alteradas^(8,12,18,20)^. La hipotensión intradialítica se asocia con mayor morbilidad, tiempo de recuperación prolongado y menor bienestar del paciente. Por lo tanto, es esencial comprender las consecuencias hemodinámicas de la alimentación intradialítica.

La fatiga es uno de los síntomas más comunes que reportan los pacientes en HD. La fatiga pos-HD, que puede durar horas y no se resuelve por completo con el descanso, afecta significativamente las actividades diarias, la participación en el tratamiento y la calidad de vida de los pacientes. Los estudios han demostrado que entre el 60 % y el 97 % de los pacientes en HD experimentan fatiga, lo que afecta significativamente su vida social y su calidad de vida^(4,5,6,21)^. Investigaciones recientes han destacado que la fatiga es un síntoma multifacético al que influyen varios factores, como la inflamación, las comorbilidades, los trastornos del sueño, el estado nutricional y el procedimiento de HD en sí mismo. Entre los factores que contribuyen a ella se encuentran la acumulación de desechos metabólicos (urea, ácido úrico y creatinina), una diálisis inadecuada, las restricciones de líquidos, las fluctuaciones de la presión arterial, la ultrafiltración excesiva, el desequilibrio electrolítico, la anemia y los factores psicológicos. La inactividad durante el tratamiento también puede provocar debilidad muscular, lo que aumenta aún más la fatiga. Además, los síntomas urémicos, como las náuseas, los vómitos y la pérdida de apetito, pueden causar desnutrición, lo que agrava la fatiga^(5,6,22)^. Aunque en la investigación nefrológica se hace cada vez más hincapié en las medidas de resultados reportadas por los pacientes, ningún estudio ha examinado directamente la relación entre la ingesta de alimentos durante la diálisis y la gravedad de la fatiga; esto representa una laguna significativa en la literatura. Por lo tanto, el presente estudio cruzado, aleatorizado y autocontrolado tuvo como objetivo evaluar los efectos de la nutrición intradialítica sobre la adecuación de la diálisis, los parámetros hemodinámicos intradialíticos y la fatiga en pacientes en HD de mantenimiento. El objetivo de la investigación es proporcionar evidencia clínica sobre las prácticas de nutrición intradialítica individualizadas mediante la integración de mediciones objetivas de la diálisis con los resultados reportados por los pacientes. En consecuencia, se evaluó la hipótesis de que la ingesta de alimentos durante la diálisis no tiene ningún efecto sobre la adecuación de la diálisis (URR y spKt/V) ni sobre los niveles de fatiga.

## MÉTODO

### Diseño del Estudio

Este estudio se diseñó como un ensayo cruzado aleatorizado, de dos períodos y dos secuencias, en el que cada participante sirvió como su propio control. La metodología cruzada se implementó de acuerdo con las consideraciones relativas al diseño cruzado en ensayos clínicos^(23)^. Además, este manuscrito se preparó de acuerdo con la extensión de 2010 de las Normas Consolidadas para el Informe de Ensayos (CONSORT) para ensayos cruzados aleatorizados^(24)^.

### Población y Criterios de Selección

Se evaluó la elegibilidad de pacientes adultos sometidos a hemodiálisis (HD) en un hospital universitario y un centro privado de diálisis en Turquía. Los criterios de inclusión fueron los siguientes: edad ≥18 años, recibir HD al menos tres veces por semana durante un mínimo de tres meses, estado clínico estable y capacidad para dar consentimiento informado. Los criterios de exclusión fueron los siguientes: infección activa, hospitalización en el mes anterior, trastornos gastrointestinales que afectaran la ingesta oral, hipotensión intradiálisis grave que requiriera intervención frecuente (PMA <65 mmHg), deterioro cognitivo, embarazo y trasplante renal programado en el futuro debido a la disponibilidad de un donante adecuado durante el período del estudio.

### Tamaño de la Muestra

La población del estudio estuvo compuesta por 127 pacientes, de los cuales 25 fueron tratados en el hospital universitario y 102 en el centro de diálisis. El tamaño de la muestra se calculó con base en el estudio de Kara y Açıkel^(25)^. En consecuencia, con un tamaño del efecto (d) de 0,71, un error de tipo I de 0,05, una potencia de 0,80 y un valor de correlación entre grupos de 0,50, se determinó que se debían incluir al menos 18 pacientes^(26)^. Sin embargo, considerando la posibilidad de que los pacientes abandonaran el estudio por cualquier motivo, se añadió una tasa de deserción del 50, y se determinó que se necesitaban al menos 36 pacientes como muestra inicial. Finalmente, se aleatorizó a un total de 36 pacientes; de ellos, 32 completaron ambos períodos del estudio y fueron incluidos en el análisis final.

### Aleatorización y Cegamiento

Los participantes fueron asignados al azar a una de dos secuencias de intervención en una proporción de 1:1 mediante una lista de asignación aleatoria generada por computadora. La secuencia A consistió en nutrición durante la diálisis en el primer período, seguida de ausencia de nutrición en el segundo período; la secuencia B consistió en ausencia de nutrición en el primer período, seguida de nutrición durante la diálisis en el segundo período. La asignación aleatoria fue realizada por un investigador independiente que no participó en el proceso de recolección de datos. Debido a la naturaleza de esta investigación, no fue posible realizar un enmascaramiento ni para los participantes ni para los investigadores.

### Intervención

Durante el período de nutrición en diálisis, los participantes consumieron una comida estandarizada una hora y media después del inicio de la diálisis. La comida aportaba aproximadamente 450 kcal y 20 g de proteína, con bajo contenido de potasio y fósforo, de acuerdo con las recomendaciones dietéticas para pacientes renales^(14,17)^. El menú de muestra incluía un huevo cocido, una rebanada de queso blanco, seis aceitunas negras sin sal o cinco aceitunas verdes, una rebanada de pan sin sal, una porción de 20 g de mermelada de fresa o de albaricoque y 200 mL de té negro. Se indicó a los participantes que consumieran toda la comida y que no consumieran ningún alimento adicional durante la diálisis. Durante el período de control, los participantes no consumieron ningún alimento durante las sesiones de diálisis, pero continuaron con su dieta habitual fuera de las sesiones. Cada período del estudio duró cuatro semanas, lo que correspondió a aproximadamente 12 sesiones de diálisis por período.

En cuanto al procedimiento de diálisis, los parámetros de HD —es decir, cuatro horas tres veces por semana, máquina Fresenius 4008s, solución de dializado que contiene glucosa de la marca Renal, dializador sintético de alto flujo de la marca Fresenius (1,7 m^2^), concentración de sodio en el dializado de 140 mEq/L, caudal sanguíneo (300–350 ml/min), caudal de dializado (500 ml/min) y temperatura del dializado (36–36,5 °C)— se mantuvieron constantes.

### Medidas de Resultado

Los resultados primarios fueron la adecuación de la diálisis, evaluada mediante la URR y el spKt/V calculados a partir de muestras de sangre de rutina tomadas antes y después de la diálisis, y la gravedad de la fatiga, que se midió al final de cada período del estudio. Los resultados secundarios incluyeron la presión arterial sistólica (PAS) y la presión arterial diastólica (PAD) intradialítica, ambas medidas al inicio del estudio y cada hora durante la diálisis; la incidencia de hipotensión intradialítica; el volumen de ultrafiltración; y los parámetros de laboratorio. El diseño de este estudio se resume en la Figura 1.

**Figura 1:** Diagrama de flujo de los participantes incluidos y analizados en el estudio, de conformidad con las Normas Consolidadas para la Presentación de Ensayos Clínicos (CONSORT) – Mersin, Turquía.

### Recolección de Datos

Los datos se recolectaron entre el 1 de abril de 2022 y el 30 de junio de 2022 por uno de los investigadores (EK), quien cuenta con 25 años de experiencia como enfermero especialista en una unidad de hemodiálisis de un hospital universitario y ha participado activamente en diversos cursos, capacitaciones y congresos científicos relacionados con la hemodiálisis. La recolección de datos se llevó a cabo utilizando tres instrumentos: el Formulario de información personal, el Formulario para monitorear la hemodiálisis y los resultados de laboratorio (Hemodialysis Monitoring and Laboratory Findings Form), y la Escala de fatiga de Piper (Piper Fatigue Scale -PFS).

El Formulario de Información Personal, desarrollado a partir de la literatura, constaba de 15 ítems que evaluaban características sociodemográficas (p. ej., edad, sexo, estado civil y nivel educativo) y variables relacionadas con la enfermedad (p. ej., etiología de la insuficiencia renal, comorbilidades, uso de antihipertensivos, medicamentos y síntomas experimentados)^(7,14,17)^.

El Formulario para monitorear la hemodiálisis y los resultados de laboratorio también se desarrolló con base en investigaciones existentes^(7,8)^. Se utilizó para recopilar datos sobre las mediciones de la presión arterial intradiálisis (previa a la diálisis, a la segunda hora y posterior a la diálisis), el volumen de ultrafiltración, el peso seco y el peso corporal previo y posterior a la diálisis. También se registraron los parámetros de laboratorio mensuales de rutina (urea, creatinina, potasio, fósforo, albúmina y hemoglobina), así como los índices de adecuación de la diálisis (spKt/V y URR)^(10,11,25)^.

La fatiga se evaluó mediante la PFS, un instrumento de 22 ítems desarrollado por Piper et al.^(27)^. Las puntuaciones totales de fatiga se calcularon como el promedio de las puntuaciones de cada ítem, donde las puntuaciones más altas indicaban una mayor fatiga percibida. El estudio de validez y confiabilidad de esta escala en Turquía fue realizado por Can et al.^(28)^, quien reportó un alfa de Cronbach de 0,94^(28)^. En el presente estudio, el coeficiente alfa de Cronbach fue de 0,92.

### Análisis y Procesamiento de Datos

Los datos recolectados se analizaron utilizando un software estadístico (IBM SPSS versión 25). Se evaluó la normalidad de las variables continuas mediante la prueba de Shapiro-Wilk. Se utilizaron pruebas t pareadas y análisis de varianza (ANOVA) de medidas repetidas para comparar dos y tres mediciones relacionadas, respectivamente, aplicándose el ajuste de Bonferroni para las comparaciones post hoc. Los datos continuos se presentan como promedios y desviaciones estándar, y los datos categóricos se presentan como frecuencias y porcentajes. Se utilizó el coeficiente de correlación de Pearson para examinar las asociaciones entre las variables continuas. La significación estadística se fijó en p < 0,05.

### Aspectos Éticos

Se obtuvo la aprobación ética del Comité de Ética en Investigación Clínica de la institución correspondiente (n.º de aprobación 199, con fecha del 23 de marzo de 2022), así como del hospital y del centro de diálisis que participaron en este estudio. Se obtuvo el consentimiento informado, tanto escrito como verbal, de todos los participantes después de que se les informara exhaustivamente sobre el propósito y los procedimientos del estudio. Además, este estudio se llevó a cabo de conformidad con los principios de la Declaración de Helsinki. El identificador de este estudio en ClinicalTrials.gov es NCT07687498.

## RESULTADOS

La edad promedio de los participantes fue de 53,7 ± 16,8 años, y la mayoría tenía 60 años o más. La hipertensión fue la causa más común de ERC, y la mayoría de los participantes padecía al menos una afección crónica adicional. La fatiga fue el síntoma reportado con mayor frecuencia (84,4 %), seguido del insomnio (62,5 %) y la debilidad (56,3 %).

### Parámetros de Laboratorio, Adecuación de la Diálisis y Volumen de Ultrafiltración

El análisis de los parámetros de laboratorio no indicó diferencias estadísticamente significativas entre los períodos de alimentación intradialítica y sin alimentación, con la excepción de los niveles séricos de fósforo (p > 0,05). Específicamente, las concentraciones séricas de fósforo fueron significativamente más altas durante el período de alimentación intradialítica (p = 0,02). Del mismo modo, no se observaron diferencias estadísticamente significativas en los parámetros de adecuación de la diálisis, incluyendo la tasa de eliminación de uracilo (URR), el spKt/V y el volumen de ultrafiltración, entre los dos períodos (p > 0,05) (Tabla 1).

**Tabla 1:** Comparación de los resultados de laboratorio y los valores de URR–spKt/V entre pacientes que ingirieron alimentos y aquellos que no lo hicieron durante la diálisis – Mersin, Turquía, 2022 (n = 32).

Variables	Con ingesta de alimentos Media ± DE	Sin ingesta de alimentos Media ± DE	Prueba t	Significación p
Potasio (mEq/L)	5,54 ± 0,68	5,36 ± 0,72	1,278	0,21
Albúmina (gr/dl)	3,76 ± 0,42	3,74 ± 0,42	0,579	0,56
Fósforo (mg/dl)	5,92 ± 1,67	5,28 ± 1,86	2,323	0,02
Hemoglobina (gr/dl)	10,44 ± 2,83	10,19 ± 2,89	0,766	0,44
URR	73,21 ± 6,37	73,43 ± 5,88	-0,217	0,83
spKt/V	1,60 ± 0,3	1,61 ± 0,25	-0,327	0,75
**Cantidad de ultrafiltración (UF)**				
Diferencia de peso entre entrada y salida	2584,38 ± 683,96	2712,5 ± 665,15	-1,339	0,19

Test t pareado, p < 0,05 (significativo).

### Presión Arterial

El análisis global de los valores de presión arterial intradialítica mostró una disminución progresiva significativa de la PAS durante las sesiones con ingesta de alimentos en comparación con aquellas sin ingesta de alimentos (p = 0.001). La PAS media en la segunda hora de diálisis fue significativamente menor durante el periodo de alimentación (118,72 ± 15,68 frente a 122,59 ± 16,22; p = 0,04). Del mismo modo, los valores de la presión arterial diastólica (PAD) en la segunda y tercera mediciones disminuyeron de forma significativa durante el periodo de ingesta de alimentos (p = 0.001), mientras que las disminuciones observadas durante el periodo sin ingesta de alimentos no fueron estadísticamente significativas (p > 0,05) (Tabla 2).

**Tabla 2:** Comparación de los valores de presión arterial entre pacientes con y sin ingesta de alimentos durante la diálisis – Mersin, Turquía, 2022 (n = 32).

Variables	Con ingesta de alimentos Media ± DE	Sin ingesta de alimentos Media ± DE	Prueba t	Significación P*
**Presión arterial sistólica**				
PAS al inicio de la diálisis	127,63 ± 13,87	127,69 ± 13,89	-0,056	0,96
PAS en la segunda hora	118,72 ± 15,68	122,59 ± 16,22	-2,089	0,04
Presión sistólica de salida de la diálisis	116,94 ± 17,56	116,94 ± 17,56	-1,081	0,29
F	23,610	20,983		
P**	<0,001	<0,001		
***1 frente a 2, 1 frente a 3, 2 frente a 3	<0,001; < 0,001; 0,41	0,002; <0,001; 0,02		
**Presión arterial diastólica**				
Presión arterial diastólica al inicio de la diálisis	80,41 ± 10,48	80,63 ± 13,43	-0,136	0,89
Presión arterial diastólica en la segunda hora	75,31 ± 10,52	76,97 ± 10,88	-1,311	0,2
Presión arterial diastólica de salida de diálisis	73,41 ± 12,65	76,41 ± 15,08	-1,212	0,24
F	23,916	2,446		
P**	<0,001	0,1		
***1 frente a 2, 1 frente a 3, 2 frente a 3	<0,001; <0,001; 0,04	–		

*Test t pareado

**ANOVA repetido

***Prueba post hoc de Bonferroni

significación p < 0,05.

### Fatiga

Al comparar los periodos con y sin ingesta de alimentos, no se encontraron diferencias estadísticamente significativas entre los grupos en cuanto a la definición de fatiga (cansancio, debilidad y fatiga mental), sus causas o estrategias de afrontamiento (p > ,05). Las sesiones de HD se señalaron como la causa más frecuente de fatiga en ambos periodos y se mencionaron con mayor frecuencia durante el periodo sin alimentación; sin embargo, la diferencia no fue estadísticamente significativa (p > 0,05). En cuanto al manejo de la fatiga, la mayoría de los participantes indicaron que realizaban actividades pasivas o de baja intensidad tras la diálisis, como descansar o dormir, ver la televisión y caminar (Tabla 3). Las puntuaciones medias de la escala PFS fueron de 106,88 ± 27,51 durante el periodo de alimentación intradialítica y de 108,63 ± 29,36 durante el periodo sin alimentación, sin que se observaran diferencias estadísticamente significativas entre ambas condiciones (p > 0,05) (Tabla 4).

**Tabla 3:** Síntomas experimentados por los pacientes, estrategias de afrontamiento y comparaciones entre grupos – Mersin, Turquía, 2022 (n = 32).

Variables	Con ingesta de alimentosn (%)	Sin ingesta de alimentosn (%)	EstadísticasPrueba de x2/valor p
Definición de fatiga*			
Cansancio	12 (57%)	9 (43%)	0,384/0,54
Debilidad	12 (50%)	12 (50%)	<0,001/0,999
Fatiga mental	9 (41%)	13 (59%)	0,659/0,42
Causas de la fatiga*			
Hipotensión	9 (60%)	6 (40%)	0,538/0,46
Sesión de hemodiálisis	10 (37,01%)	17 (62,9%)	1,633/0,20
Afecciones relacionadas con la anemia	3 (37,5%)	5 (62,5%)	0,412/0,52
Vehículo de transporte para hemodiálisis	11 (61,1%)	7 (38,9%)	0,789/0,37
Dolor crónico	2 (33,3%)	4 (66,6%)	0,499/0,48
Cómo afrontar el síntoma de fatiga*			
Dormir/descansar	13 (65%)	7 (35%)	1,569/0,21
Dar un paseo	10 (55,5%)	8 (44,5%)	0,203/0,65
Ver la televisión/Escuchar música	13 (61,9%)	8 (38,1%)	1,072/0,30
Cuidar de las plantas	4 (33,3%)	8 (66,6%)	1,098/0,29
Viajar con amigos o familiares	5 (55,5%)	4 (44,4%)	0,097/0,76
Cuidar de animales	3 (33,33%)	6 (6,6%)	0,798/0,37
Lectura	8 (57,14%)	6 (42,85%)	0,260/0,61

*Se marcó más de una opción.

**Tabla 4:** Comparación de las puntuaciones medias de la Escala de Fatiga de Piper (PFS) entre pacientes con y sin ingesta de alimentos durante la diálisis – Mersin, Turquía, 2022 (n = 32).

Variables	Con ingesta de alimentos Media ± DE	Sin ingesta de alimentos Media ± DE	Prueba t	Significación p
SLP	106,88 ± 27,51	108,63 ± 29,36	-0,409	0,69

Prueba t para muestras emparejadas, nivel de significación p < 0,05.

## DISCUSIÓN

En este estudio cruzado aleatorizado, la ingesta nutricional intradialítica no afectó significativamente los parámetros de adecuación de la diálisis, incluidos la URR y el spKt/V, ni alteró la gravedad de la fatiga. Sin embargo, la nutrición intradialítica se asoció con una disminución más pronunciada de la presión arterial sistólica (PAS) y la presión arterial diastólica (PAD) durante las sesiones de diálisis, particularmente durante la fase media del tratamiento. Estos hallazgos brindan información clínicamente relevante tanto sobre los posibles beneficios como sobre los riesgos de la ingesta de alimentos durante la hemodiálisis (HD) y respaldan un enfoque más individualizado de las prácticas nutricionales intradialíticas.

En este estudio, se observó que los niveles séricos de fósforo eran significativamente más altos cuando se consumían alimentos durante la HD. Estudios previos han informado que la nutrición intradialítica puede contribuir a la hiperfosfatemia debido al contenido de fósforo de las comidas^(11,20)^. Sin embargo, en el presente estudio, los niveles de fósforo se mantuvieron dentro de límites clínicos aceptables, probablemente debido a la composición estandarizada de las comidas y al tratamiento simultáneo con quelantes de fosfato. Este hallazgo sugiere que las comidas cuidadosamente planificadas con una restricción adecuada de fósforo pueden reducir los posibles riesgos metabólicos cuando se implementa la nutrición intradialítica.

Los resultados de este estudio indican que la ingesta de nutrientes intradialítica no compromete la adecuación de la diálisis. Este hallazgo concuerda con estudios previos que reportaron cambios mínimos o clínicamente no significativos en la URR o el spKt/V cuando las comidas se estandarizaron y las prescripciones de diálisis se mantuvieron sin cambios^(6,7,10,11,13)^. Rao et al. demostraron que la nutrición intradialítica no reducía significativamente la eliminación de urea en comparación con la alimentación predialítica, particularmente cuando se controlaban la composición y el momento de las comidas^(7)^. Setiovati et al. afirmaron que el momento más adecuado para la alimentación intradialítica es la fase tardía de la sesión de diálisis. Específicamente, informaron que la alimentación durante la primera y la segunda hora provocó cambios hemodinámicos más significativos en la presión arterial sistólica (PAS) y la presión arterial media (PAM), mientras que la alimentación hacia el final de la sesión se asoció con una respuesta hemodinámica más estable y una mejor preservación de la adecuación de la diálisis. Sin embargo, algunos estudios han informado que el consumo de alimentos durante la diálisis puede reducir la adecuación de la diálisis; por lo tanto, no recomiendan la alimentación intradialítica^(8,18,20)^. Kara y Açıkel^(25)^ encontraron una disminución significativa en los parámetros de adecuación de la diálisis entre los pacientes que consumían alimentos durante la diálisis, lo que sugiere que la vasodilatación esplácnica y la distribución alterada del flujo sanguíneo podrían afectar el aclaramiento de solutos. Estas inconsistencias en la investigación existente pueden explicarse por diferencias en el tamaño de las comidas, la composición de macronutrientes y la tecnología de diálisis. Es importante destacar que el presente estudio utilizó un diseño cruzado autocontrolado, lo que minimiza la variabilidad interindividual y mejora la validez interna. Además, los participantes presentaban una competencia de diálisis basal relativamente alta, lo que podría haber limitado la capacidad de detectar pequeñas disminuciones en la URR o el spKt/V. Estos hallazgos respaldan la opinión cada vez más extendida de que las medidas de adecuación de la diálisis por sí solas podrían no reflejar plenamente el impacto clínico de las intervenciones intradialíticas sobre la vasodilatación esplácnica^(10,12,13)^. Dado que los pacientes pasan mucho tiempo en las sesiones de hemodiálisis, permitirles elegir sus alimentos favoritos puede hacer que estas sesiones les resulten más llevaderas y mejorar su adherencia al tratamiento^(13,14,29)^. El enfoque más adecuado sería abordar el estado nutricional de los pacientes con estrategias individualizadas basadas en sus factores de riesgo, especialmente en el caso de los pacientes propensos a la neuropatía autonómica y a la hipotensión sintomática, en consulta con un nutricionista.

El hallazgo clínico más notable de este estudio fue la mayor reducción de la presión arterial observada durante el período de alimentación. La literatura presenta resultados contradictorios con respecto al efecto de la nutrición intradialítica sobre la hipotensión intradialítica. Algunos estudios han informado que la nutrición intradialítica puede aumentar la inestabilidad hemodinámica^(8,12,15,16,18,19)^. Sin embargo, en otros estudios no se observó un aumento significativo en el riesgo de hipotensión cuando se contó con una selección adecuada de pacientes y prácticas de nutrición controladas^(9,10,11,12,13,14,15,22)^. A modo de ejemplo, Hou et al.^(30)^ señalaron que la hipotensión intradialítica se asocia con un bajo nivel de función física y que el rendimiento físico se ve más afectado en pacientes que experimentan hipotensión frecuente; por el contrario, Usakli et al.^(31)^ informaron que la nutrición intradialítica aumentó la fuerza de prensión manual y los niveles séricos de albúmina, mientras que Choi et al. demostraron que las comidas ricas en proteínas no aumentaron la frecuencia de la hipotensión intradialítica sintomática y que los pacientes tenían una actitud positiva hacia la nutrición durante la diálisis^(13)^. Estos hallazgos sugieren que, a pesar de los posibles riesgos hemodinámicos de la nutrición intradialítica, esta puede brindar beneficios nutricionales y funcionales siempre y cuando se garantice una selección adecuada de los pacientes y un monitoreo minucioso. Desde el punto de vista de la enfermería, estos hallazgos resaltan la importancia de un monitoreo vigilante de la presión arterial intradialítica, una evaluación individualizada del riesgo y una selección cuidadosa de los pacientes al implementar intervenciones nutricionales intradialíticas.

La fatiga siguió siendo muy prevalente (84 %) en este estudio, y la mayoría de los participantes identificaron el procedimiento de HD en sí mismo como un factor contribuyente importante. Muchos participantes informaron que sobrellevaban la fatiga después de la diálisis descansando, durmiendo o realizando actividades de baja intensidad. Estos hallazgos concuerdan con los de estudios previos^(4,5,6,21,22,29)^, lo que demuestra que la fatiga es uno de los síntomas más agobiantes entre los pacientes que reciben HD de mantenimiento. You et al.^(32)^ encontraron que el 71,3 % de los pacientes de su estudio experimentaban fatiga. En el estudio de Bossola et al.^(5)^, los síntomas emocionales (sentirse triste, sentir ansiedad y tener dificultades para concentrarse) fueron significativamente más frecuentes en los pacientes con fatiga. Casaux-Huertas et al.^(22)^ informaron que un estado nutricional deficiente en pacientes en HD se asocia con fatiga y trastornos del sueño, y enfatizaron que un apoyo nutricional adecuado, especialmente la nutrición intradialítica, puede ofrecer beneficios potenciales en términos de reducir la carga de los síntomas y mejorar la calidad de vida. Sin embargo, la nutrición intradialítica no modificó significativamente los índices de fatiga. La fatiga en los pacientes en hemodiálisis es un síntoma complejo y multifactorial en el que influyen la anemia, la inflamación, los trastornos del sueño, las comorbilidades, la depresión, el estrés relacionado con la diálisis y los factores psicosociales^(4,5,6,22,30,32)^. Por lo tanto, una intervención nutricional a corto plazo por sí sola podría ser insuficiente para producir mejoras medibles en la gravedad de la fatiga. No obstante, la ausencia de un empeoramiento de la fatiga sugiere que la nutrición intradialítica podría ser tolerada desde el punto de vista de los síntomas en pacientes adecuadamente seleccionados.

En general, los hallazgos actuales respaldan un enfoque personalizado de la nutrición intradialítica en lugar de una restricción universal o una implementación rutinaria. Para los pacientes clínicamente estables con una ingesta nutricional deficiente, las comidas cuidadosamente planificadas durante la diálisis podrían ser beneficiosas. Por el contrario, los pacientes con hipotensión recurrente, disfunción autonómica o inestabilidad cardiovascular significativa pueden requerir un manejo más cauteloso. La colaboración entre enfermeras, nefrólogos y dietistas renales es esencial para optimizar la seguridad del paciente y el beneficio nutricional^(6,7,10,14,15,16,17)^. Además, estos resultados concuerdan con las recomendaciones recientes de expertos que abogan por la toma de decisiones individualizadas con respecto a la ingesta de alimentos durante las sesiones de HD, en lugar de políticas universales.

También deben reconocerse varias limitaciones de este estudio. En primer lugar, el tamaño relativamente pequeño de la muestra podría haber limitado la potencia estadística para detectar diferencias sutiles en los resultados de la adecuación de la diálisis y la fatiga. En segundo lugar, aunque el diseño cruzado redujo la variabilidad interindividual, la ausencia de un período de lavado podría haber introducido efectos residuales de arrastre; sin embargo, los efectos fisiológicos agudos de la ingesta de alimentos durante la diálisis suelen ser de corta duración. En tercer lugar, dado que la fatiga se evaluó mediante un instrumento de autoinforme, la percepción subjetiva y el sesgo de respuesta podrían haber influido en los datos recopilados. Además, este estudio se llevó a cabo en dos centros utilizando un protocolo de comidas estandarizado, lo que puede limitar la generalizabilidad de los hallazgos a otros entornos con características de pacientes o prácticas nutricionales diferentes. Por último, el período de seguimiento relativamente corto limita las conclusiones sobre los efectos a largo plazo de la nutrición intradialítica. Por lo tanto, los hallazgos deben interpretarse teniendo en cuenta estas consideraciones.

## CONCLUSIÓN

Este estudio aleatorizado cruzado demostró que la nutrición intradialítica no comprometió significativamente la adecuación de la diálisis ni empeoró la gravedad de la fatiga en pacientes sometidos a HD de mantenimiento. Sin embargo, se observaron mayores reducciones en la presión arterial sistólica (PAS) y la presión arterial diastólica (PAD) tras la ingesta de alimentos durante la diálisis, lo que resalta la importancia de monitorear de manera minuciosa la presión arterial durante la administración nutricional. Estos hallazgos contribuyen a la literatura al sugerir que la nutrición intradialítica puede ser un enfoque clínicamente viable cuando se adapta a la tolerancia y los perfiles de riesgo individuales de cada paciente, en lugar de restringirse de manera universal o recomendarse de forma rutinaria. Es importante destacar que este estudio también ofrece nuevos conocimientos sobre la relación entre la nutrición intradialítica y la fatiga, un área que aún no se ha investigado lo suficiente. Desde una perspectiva clínica, los hallazgos resaltan la importancia de la colaboración multidisciplinaria entre enfermeras, nefrólogos y dietistas renales al desarrollar estrategias nutricionales para las sesiones de diálisis. No obstante, se necesitan estudios multicéntricos más amplios que evalúen diferentes tipos de composición nutricional y resultados a largo plazo para aclarar aún más la seguridad y el impacto clínico de la nutrición intradialítica.

## Data Availability

El conjunto completo de datos que respalda los resultados de este estudio se publicó en el propio artículo.
